# Path planning for manipulators based on the planar constraint RRT* algorithm

**DOI:** 10.1038/s41598-026-44581-7

**Published:** 2026-03-23

**Authors:** Minyue Li, Wensong Jiang, Zai Luo, Yan Wang, Li Yang

**Affiliations:** 1 The College of Metrology Measurement and Instrument, China Jiliang University, Hangzhou, 310018 PR China; 2 The Department of Information Engineering, China Jiliang University, Hangzhou, 310018 PR China

**Keywords:** Path planning, Collision avoidance, RRT*, Planar constraints, Manipulator, Engineering, Mathematics and computing

## Abstract

Path planning is crucial for automatic measurement to ensure a collision-free process for manipulators. However, the more complex the measurement environment, the more complex the path planning scheme is often required in order to meet the above requirements. To overcome this problem, a planar constraint RRT* method (PC-RRT*) is proposed to limit nodes to a specific plane, reduce the blindness of RRT*, and smooth paths. The measurement space model is built according to the position of the manipulator, the obstacle environment, the starting point, and the ending point. A set of equiangular interval collinear planes is built by utilizing the starting and ending line as the central axis. A local path on each plane is planned by extended rapidly exploring random tree strategy. By optimizing the planned local path set, the high-quality measurement path will be solved. Both numerical simulation and experimental analysis are carried out to verify the effectiveness of the PC-RRT* method. The experimental results show that the average path lengths (APL) are 216.03 mm for PC-RRT*, 346.83 mm for RRT, 241.93 mm for RRT*, and 383.6 mm for Q-learning (QL), the average number of nodes is 9.5 for PC-RRT*, 11.12 for RRT, 11.6 for RRT*, and 18.92 for QL. The suggested PC-RRT* has an 37.71% improvement for APL and 14.57% improvement for path nodes compared to the RRT algorithm. PC-RRT* has a 10.71% improvement for APL and 18.1% improvement for path nodes compared to the RRT* algorithm. Additionally, PC-RRT* achieves a 43.68% improvement for APL and a 49.79% improvement for path nodes compared to the QL algorithm. In all, the PC-RRT* method is superior to other traditional methods.

**Supplementary Information:**

The online version contains supplementary material available at 10.1038/s41598-026-44581-7.

## Introduction

Path planning is essential to automatic measurement with collision-free, which is widely applied to measure auto body parts, aircraft skin, ship hull, and other complex geometry^1–3^. The study of path planning problems in complex environments is a crucial aspect in the field of robot control technology^4^. However, such complex structures with a challenging measurement environment require an accurate and collision-free path planning scheme, which is hard to achieve.

The path planning algorithm can be divided into local and global path planning algorithms. The local path planning algorithms are mainly applied to unknown environments by real-time collecting the environmental information, such as the artificial potential field method^5^, dynamic window method, and fuzzy algorithm^6,7^. The global path planning algorithms are generally applied to environments with known obstacle information, such as the graph search method, sampling-based method, and intelligent optimization method^8^.

The Graph search method includes Dijkstra algorithm^9^, A* algorithm^10^, and its modified form. For example, J.Y. Liu proposed the (CDT)-Dijkstra algorithm to reduce the path planning state space from 2D to 1D^11^. D. Hong presented An Accelerated Dijkstra algorithm specifically designed for medical image segmentation. This method can run image segmentation successfully, has fewer interactive times, and runs faster than the Classical Dijkstra Algorithm^12^. X.Y. Zhao proposed an adaptive A* algorithm to shorten the path with few inflection points by selecting more reasonable preselect points^13^. J. Li integrated an enhanced R5DOS crossover model with an improved A* algorithm to reduce computation times and minimize node visits^14^. Anit Kumar et al. proposed a hybrid algorithm combining the A* algorithm and the visibility graph algorithm to address the robot path planning problem, resulting in shorter paths^15^. Y.L. Yang proposed the AAPF* algorithm to enhance the safety of autonomous vehicles and facilitate smoother global path planning^16^. However, in high-dimensional spaces and complex environments, modeling the measuring obstacles becomes challenging for graph search algorithms; a small mistake may decrease the efficiency of path planning^17^.

The Intelligent optimization method draws inspiration from biological behaviors, which include the neural network method, genetic algorithm^18^, ant colony algorithm, and particle swarm algorithm^19,20^. The neural network algorithm is suitable for scenarios with large energy consumption^21,22^. J.L. Yu introduced a neural network path planning model for mobile robots using hierarchical reinforcement learning (RL), achieving smooth paths and demonstrating strong generalization across scenarios^23^. A novel cooperative planning architecture is proposed, integrating a graph neural network with a task-oriented knowledge fusion sampling method to aggregate knowledge from a specific orientation, ensuring effective and stable training^24^. Sebastian Mihai Ardelean proposed the Quantum Genetic Algorithm (QGA), an innovative approach that integrates genetic programming with quantum computing to address search and optimization problems^25^. P.P. Wu proposed an enhanced ant colony optimization algorithm that utilizes two distinct sets of ants operating from opposite ends to compute paths. This approach facilitates optimal path determination upon convergence, thereby improving the overall efficiency and effectiveness of the algorithm^26^. Rehan Ali Khan proposes a generalized particle swarm optimization (GPSO) algorithm that enhances standard PSO by incorporating individual extreme information from all particles, improving optimization performance and ensuring better search capabilities through increased information exchange^27^. In summary, intelligent optimization methods often suffer from slow convergence rates and the risk of falling into local optima, which constrains their effectiveness in high-dimensional spaces and complex environments.

The random sampling method is based on random sampling of space points to search for connectivity paths^28^. Compared to traditional path planning algorithms, it can identify feasible paths and handle high-dimensional, complex cases without requiring complex environment modeling^29^. To overcome the low smoothness, blind search, low convergence speed, and other limitations, the RRT algorithm is enhanced by a model-based method. For example, S.J. Lei proposed the MSF-RRT algorithm, which enhances RRT by combining target bias, bias expansion, and adaptive step size strategies, optimizing path efficiency and length^30^. H.C. Ji proposed an ellipsoidal fast extended random tree algorithm (E-RRT*) that showed better performance in terms of path length and smoothness^31^. Francesco Grothe proposed the ST-RRT* algorithm (Spatial-Temporal Asymptotically Optimal Bidirectional Motion Planning) to optimize both the initial solution time and the final solution cost^32^. Ahmed Hussain Qureshi proposed the IB-RRT* algorithm, utilizing a bidirectional tree method and incorporating an intelligent sample insertion heuristic. This approach ensures nearly certain convergence to the optimal path solution and facilitates rapid convergence^33^. Y. Wang proposed a direction-guided fast exploration random tree algorithm (DG-RRT) that reduces both the APL and average planning time by adjusting the step size, optimizing threshold parameters, and smoothing curves^6^. Y. Liu proposed an efficient method for boundary exploration utilizing a double-layer rapid-exploration random tree (DL-RRT), which effectively reduced both the path planning time and the total path length^34^. Y. Huang proposed the TRBi-RRT algorithm to provide a viable solution for clinical applications, outperforming conventional methods in computational speed, path representation, and search capabilities^35^. J. Wang proposed an improved RRT* algorithm, FC-RRT*, utilizing prior knowledge of the task to expand the path tree bidirectionally from both the starting point and the target point. This approach allows for obtaining a better initial path more quickly^36^. B.P. Wang proposes the CAF-RRT* algorithm for two-dimensional path planning, which combines Quick-RRT* and bidirectional RRT to improve path efficiency and smoothness through a circular arc fillet method^37^. L.F. Jia et al. proposed the MDA-RRT series of algorithms for path planning of hyper-redundant manipulators, which converts the joint deflection angle constraint into a path deflection angle constraint via geometric transformation, thereby ensuring feasible motion without increasing computational load^38^.

For the measuring manipulator, when the measurement scenario is fixed, a shorter measurement path with fewer nodes is more advantageous than reducing the algorithm planning time. To achieve this, we propose an approach that approximates the three-dimensional path into two -dimensional using graph search algorithms, providing a high-quality solution. The planar constraint RRT* (PC-RRT*) method is proposed to limit the nodes on a certain plane, reduce the indeterminacy of the RRT*, shorten the path length, and smooth paths. Both numerical simulations and experimental analysis are carried out to verify the suggested method.

The contributions of this paper are summarized as follows:

1. PC-RRT* algorithm restricts nodes to a specific plane, reducing the uncertainty in RRT* node generation. This approach effectively reduces the path length and the associated standard deviation, enhances efficiency, and conserves energy.
2. The planar constraint sampling method utilizes the Rodrigues’ rotation formula to explore multiple planes. This approach identifies the optimal plane in three-dimensional space by constraining randomly generated nodes to a single plane. To ensure comprehensive coverage, consistent angular intervals are established to explore the entire 360-degree range on that plane. This method is also applicable to other 3D algorithms, providing an effective approach to improving planning efficiency.
3. Based on multiple simulation experiments, the PC-RRT* algorithm ultimately employs quartic polynomial fitting, effectively enhancing path smoothness, reducing joint wear, and extending the robot arm’s service life. Furthermore, it helps mitigate the risk of manipulator singularity.
4. Simulations in diverse environments, along with real-world experiments, verify the feasibility of the proposed method and demonstrate its superior performance compared to other methods.

### Overview of the planar constraint PC-RRT* method

The PC-RRT* algorithm is a path planning method in three-dimensional space that consists of two parts. The first part involves establishing a new coordinate system based on the position of the starting point through coordinate transformation. The second part includes four steps: planning a path within the plane under the new coordinate system, rotating the constrained plane in a polar coordinate manner outside the plane, performing path selection in three-dimensional space, and generating the final high-quality path. The specifics are as follows:

1. Transformation of the spatial coordinate system

**Fig. 1:**
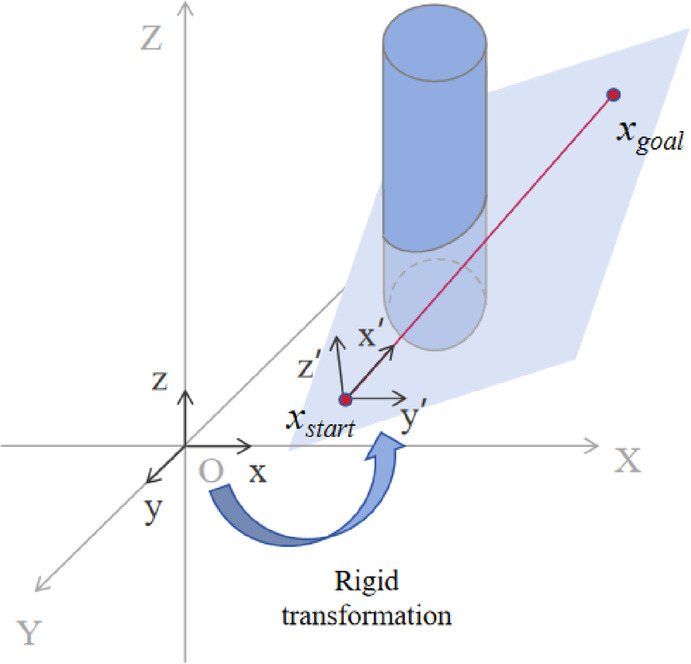
Coordinate transformation schematic diagram.

The existing coordinate system is XOY. Through coordinate transformation, X′O′Y′ is obtained, where the origin O′ represents the coordinates of the starting point, and the axis connecting the starting point and the endpoint serves as the X′ axis. The three points formed by the starting point, the endpoint, and any arbitrary point in three-dimensional space define a plane. The Z′ axis is perpendicular to this plane and passes through the starting point, which is the origin. At this point, the two axes are determined. The third axis, Y′, is defined using the right-handed coordinate system. Thus, the rotated coordinate system is established. A specific schematic is shown in Fig. 1.

2. Path planning method
  1. Planar-constrained path planning

**Fig. 2:**
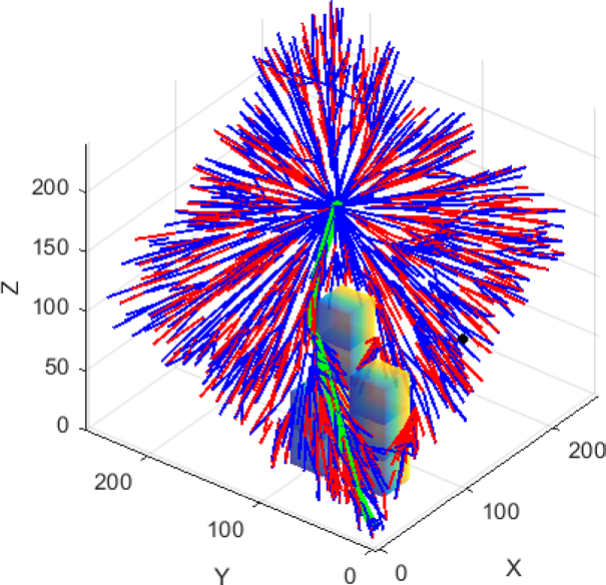
Path planning schematic under planar constraints.

The starting point, the goal point, and any arbitrary point in three-dimensional space define a constrained plane. Once this constrained plane is established, path planning is conducted within it (Supplmentary Information). A schematic is shown in Figure Fig. 2.

(2) Rotation-constrained plane

**Fig. 3:**
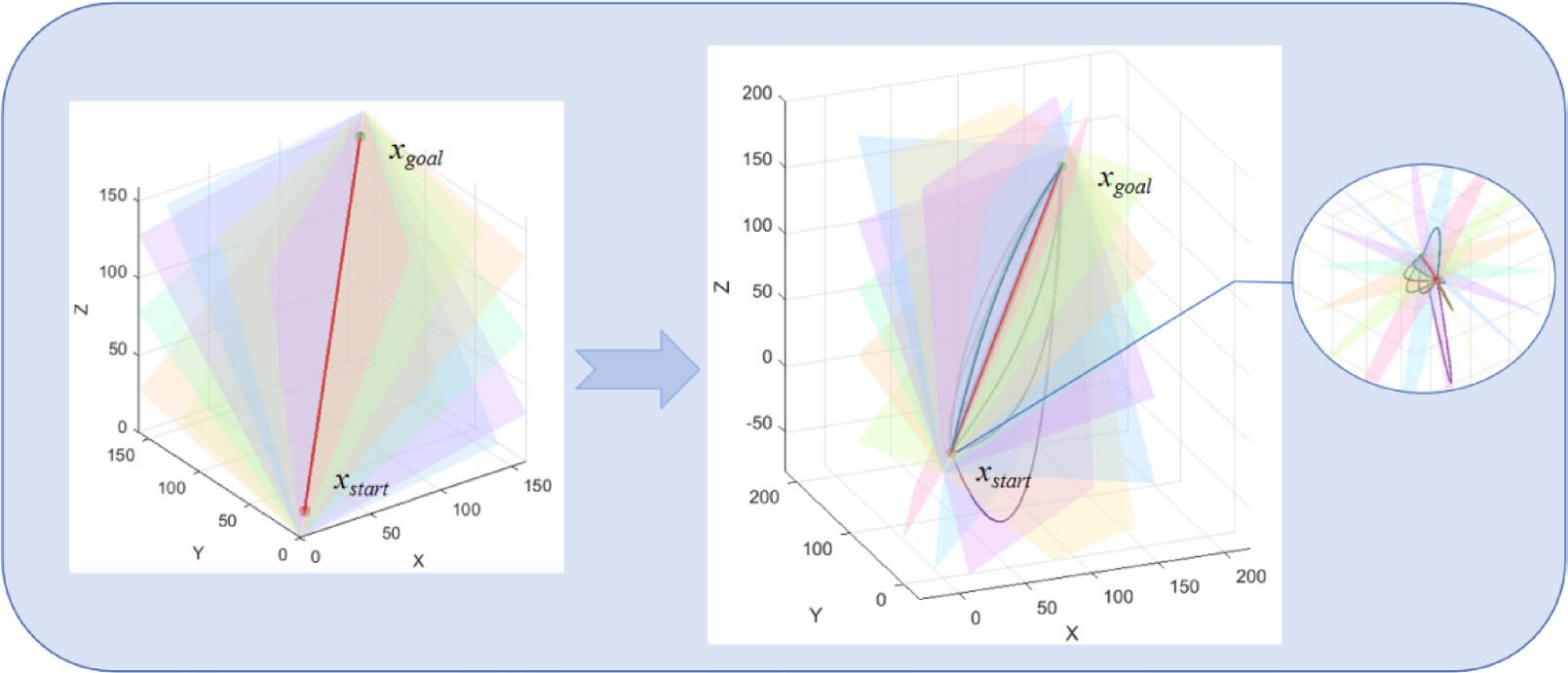
Schematic diagram of the rotated planes.

The establishment of a constrained plane is followed by its rotation at specified angles using Rodrigues’ rotation formula, with path planning executed on each resulting plane. The specific schematic is provided in Fig. 3, where the red point denotes the starting point (*x*_*start*_), the green point represents the goal point (*x*_*goal*_), and the red line segment indicates the axis connecting the starting point and the goal point. An arbitrary point is selected in the three-dimensional space outside of this axis, and together with the axis, these define a plane. This plane is then rotated around the axis at designated angles to generate multiple distinct planes. The differently colored planes in the figure represent those associated with various rotation angles. The curves illustrated in the right diagram depict the path planning conducted from the starting point to the goal point across different planes corresponding to these angles. To enhance the clarity of the schematic, obstacle information has been omitted.

(3) Spatial high-quality path decision

For path planning of the measuring manipulator, when the obstacle environment is fixed, the system will perform multiple repeated measurements on a specific object to be tested, requiring only a single path planning. In this context, a shorter path length and a reduced number of nodes are prioritized over computational efficiency in planning. In the path planning for measurement manipulators, the planning time, path length, and number of path nodes should be considered comprehensively. A weighted comprehensive evaluation method is adopted for these three metrics with the following weight configuration: time weight *w*_*t*_ = 0.1, length weight *w*_*l*_ = 0.6, and node weight *w*_*n*_ = 0.3. The comprehensive evaluation metric is defined by the following formula:

1 $$E{\text{= }}{w_t} * Nor{m_t}+{w_l} * Nor{m_l}+{w_n} * Nor{m_n}$$

2 $$Nor{m_t}=\frac{{t - {t_{\hbox{min} }}}}{{{t_{\hbox{max} }} - {t_{\hbox{min} }}}}\,,\,Nor{m_l}=\frac{{l - {l_{\hbox{min} }}}}{{{l_{\hbox{max} }} - {l_{\hbox{min} }}}},\,\,Nor{m_n}=\frac{{n - {n_{\hbox{min} }}}}{{{n_{\hbox{max} }} - {n_{\hbox{min} }}}},$$

where *Norm*_*t*_, *Norm*_*l*_, and *Norm*_*n*_ represent the normalized values of the time, path length, and path node metrics, respectively. A lower value of *E* indicates superior comprehensive performance of the algorithm.

(4) High-quality path generation

Based on the comprehensive evaluation model constructed in the spatial high-quality path decision, the system evaluates and ranks all candidate paths obtained within the rotated planes. The final outputted high-quality path is the one with the lowest comprehensive evaluation value (*E*) among all planes. This path achieves the best balance among planning time, path length, and smoothness while satisfying the three-dimensional obstacle avoidance constraints. Additionally, multiple paths with closely scored E-values can be output together as alternative paths.

### Modeling of planar constraint PC-RRT* method

This section will explore the equidistant plane constraint model, which is employed to regulate the distribution of random sampling points within a plane during the path planning process. An improved method is proposed, where randomly generated nodes are confined to a single plane. The specific procedure is as follows: A reference plane is established based on the straight-line distance between the starting point and the target point. The reference plane is then rotated at specified angular intervals to identify the optimal plane. The selection of the optimal plane prioritizes obstacle avoidance and minimizes path length. The method uses the Rodrigues’ rotation formula to rotate the constrained plane around the line connecting the start and target points.

**Fig. 4:**
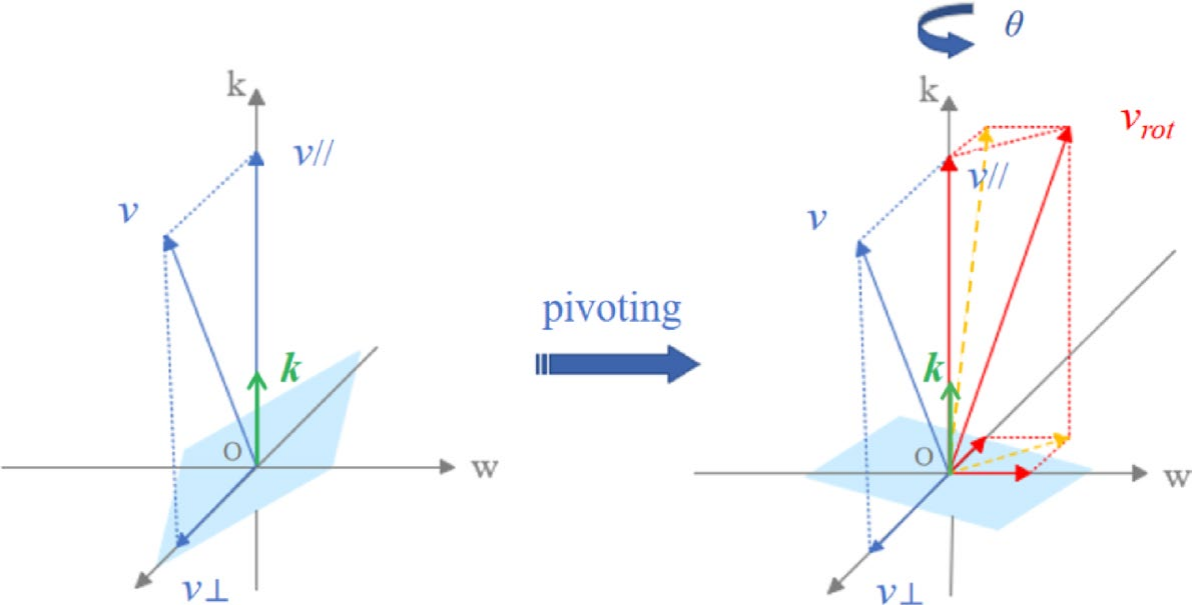
Schematic diagram of Rodrigues’ rotation formula.

Rodrigues’ rotation formula is described as follows: as shown in Fig. 4, any vector ***v*** in three-dimensional space rotates $\theta$ angle about the unit vector ***k*** in the same direction as the axis of rotation. $\theta$ is the Angle through which ***v*** rotates counterclockwise around *k* to find the rotated vector *v*_*rot*_^40^.

The vector ***v*** will be divided into perpendicular components${\boldsymbol{v}} \bot$and parallel components${\boldsymbol{v}}\parallel$, with${\boldsymbol{v}}={\boldsymbol{v}} \bot +{\boldsymbol{v}}\parallel$. The following can be obtained from the expansion of the vector triple integral:

3 $${\boldsymbol{v}} \bot = - {\boldsymbol{k}} \times \left( {{\boldsymbol{k}} \times {\boldsymbol{v}}} \right)$$

4 $${{\boldsymbol{v}}_{rot}} \bot =\frac{{{\boldsymbol{v}} \bot }}{{\parallel {\boldsymbol{v}} \bot \parallel }} \cdot \parallel {\boldsymbol{v}} \bot \parallel \cos \left( \theta \right)+\frac{{{\boldsymbol{k}} \times {\boldsymbol{v}}}}{{\parallel {\boldsymbol{k}} \times {\boldsymbol{v}}\parallel }} \cdot \parallel {\boldsymbol{k}} \times {\boldsymbol{v}}\parallel \sin \left( \theta \right)$$

where Eq. (4) can be synthesized and simplified into matrix form as follows:

5 $${{\boldsymbol{v}}_{rot}}={\boldsymbol{v}}+\left( {1 - \cos \left( \theta \right)} \right) \cdot \left[ {\left( {{\boldsymbol{k}} \times {\boldsymbol{v}}} \right) \cdot {\boldsymbol{k}} - \left( {{\boldsymbol{k}} \times {\boldsymbol{k}}} \right) \cdot {\boldsymbol{v}}} \right]+{\boldsymbol{k}} \times {\boldsymbol{v}} \cdot \sin \left( \theta \right)$$

The following can be obtained by expanding it with a triple integral:

6 $${{\boldsymbol{v}}_{rot}}={\boldsymbol{v}}+\left( {1 - \cos \left( \theta \right)} \right) \cdot {\boldsymbol{k}} \times \left( {{\boldsymbol{k}} \times {\boldsymbol{v}}} \right)+{\boldsymbol{k}} \times {\boldsymbol{v}} \cdot \sin \left( \theta \right)$$

7 $$M=I+\left( {1 - \cos \left( \theta \right)} \right) \cdot \mathop R\nolimits_{K}^{2} +\mathop R\nolimits_{K} \cdot \sin \left( \theta \right)$$

8 $${{\boldsymbol{v}}_{rot}}=M \cdot {\boldsymbol{v}}$$

The rotation axis ***k*** is defined by connecting the starting point and the target point, and then a point is chosen arbitrarily in three-dimensional space. A plane is defined by three points, from which the plane equation and the normal vector can be determined. Rodrigues’ rotation formula is used to rotate the normal vector ***v*** around the rotation axis ***k*** by a certain $\theta$, calculate the normal vector after rotation, and then transform the normal vector after rotation into a plane equation. This plane equation is taken as one of the constraints of the RRT* algorithm, restricting the randomly generated points to lie on the same plane.

### Planar constraint RRT* method

**Fig. 5:**
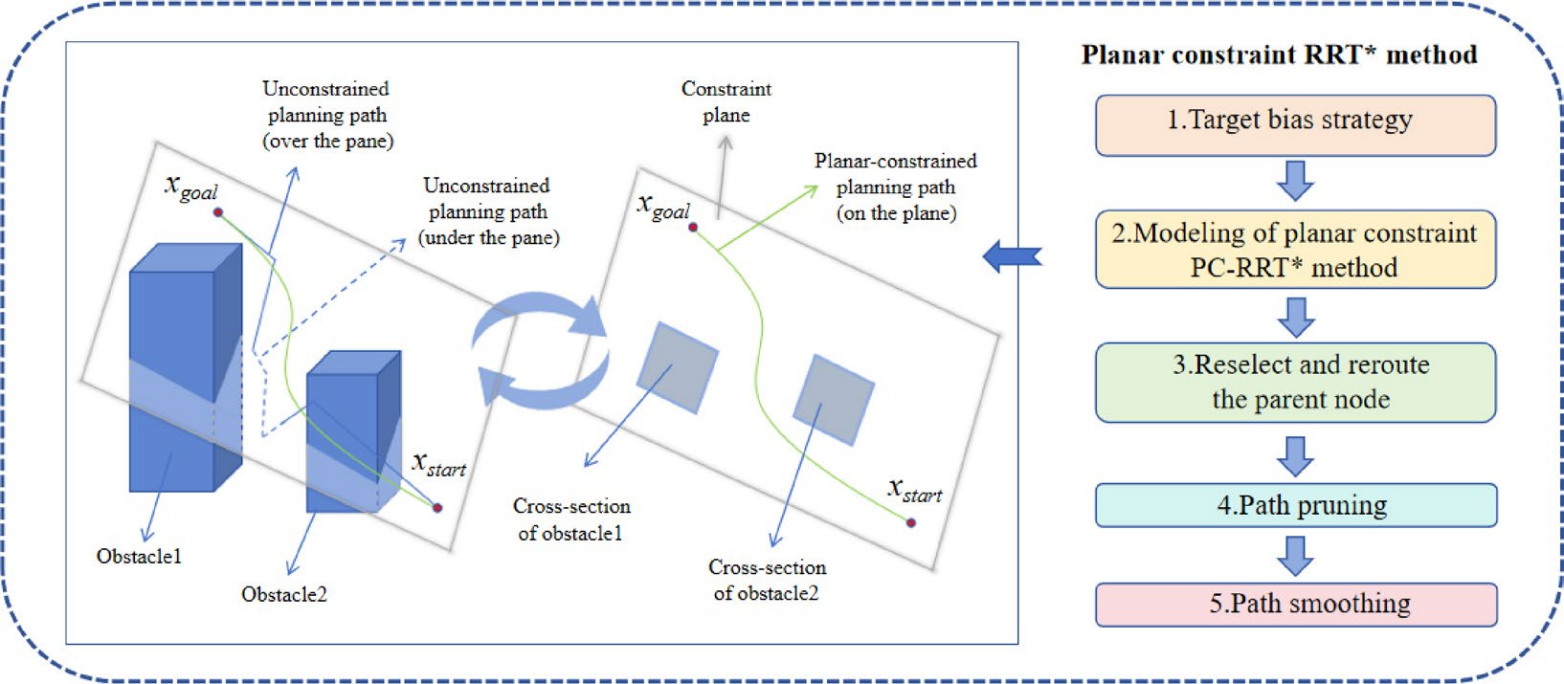
Principle diagram of PC-RRT* algorithm.

Building on the equidistant plane constraint model introduced in the previous section, this section discusses the PC-RRT* path planning algorithm. This includes the target bias strategy, re-selecting parent nodes, and rerouting, path pruning, and smoothing. The schematic diagram of the PC-RRT* algorithm is shown in Fig. 5.

The PC-RRT* algorithm consists of five major components: target bias strategy, planar constraint modeling, parent node reselection and rerouting, path pruning, and path smoothing. The small diagram on the left shows a three-dimensional view of a planar cutting obstacle, where the blue line represents the path without planar constraints (with the dashed blue line below and the solid blue line above the constraint plane). On the right, the sectional view of the planar cutting obstacle is depicted, with the green line representing the path with planar constraints and smoothing applied. Clearly, the green path is shorter and smoother than the blue path.

### Target bias strategy

To improve target guidance and algorithm convergence, this paper incorporates target bias into PC-RRT* to direct the sampling process. The target bias probability *p* (0 ≤ *p* ≤ 1) is compared with a random number (0 < *rand* < 1). If *rand* < *p*, the random point is *x*_*goal*_; if *rand* ≥ *p*, a random function generates a 3D coordinate sampling pool constrained to specified plane constraints. This ensures that the random tree expansion targets the goal and stays on the plane, reducing search indeterminacy in RRT*. For scalability and effective target-constrained sampling, the value of *p* is set to 0.5. The sampling process is as follows.

9 $${x_{rand}}=\left\{ {_{{R ,\, rand\,\, \geqslant p}}^{{{x_{goal}} ,\, rand\,<\,p}}} \right.$$

10 $${x_{goal}}=\left( {{x_g},{y_g},{z_g}} \right)$$

11 $${R_i}=\left( {{R_i}x,{R_i}y,{R_i}z} \right)$$

12 $${d_i}=\sqrt {{{\left( {{x_g} - {R_i}x} \right)}^2}+{{\left( {{y_g} - {R_i}y} \right)}^2}+{{\left( {{z_g} - {R_i}z} \right)}^2}}$$

where *i* refers to the *i*^*th*^ random sample. When *d*_*i*_ is minimized, *R* = *R*_*i*_. Random sampling points must conform to the plane equation constraints, i.e.

13 $$\sqrt {A \times {R_i}x+B \times {R_i}y+C \times {R_i}z+D} <\,V$$

where the theoretical value of *V* is 0. For programming convenience, the value of *V* is set to 0.01. *A*, *B*, *C*, and *D* are the plane coefficients obtained by rotating the reference plane using the Rodrigues’ rotation formula.

Multiple random samples should be obtained, and location information substituted into Eq. (13) to evaluate condition satisfaction. If a point meets the distance criteria, sampling is complete. Otherwise, iterative sampling continues until a valid point is found. The primary method is outlined in Algorithm 1.

**Algorithm 1:**
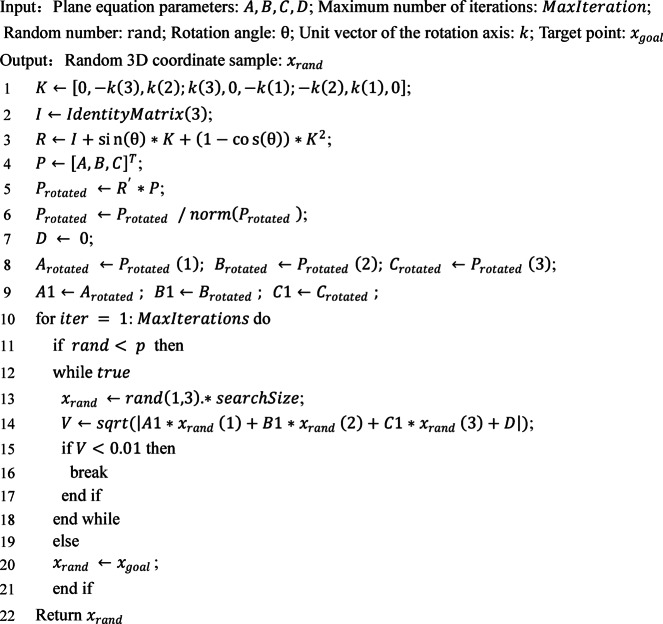
Planar-constrained point sampling.

### Reselect and reroute the parent node

Before delving into the PC-RRT* algorithm, a brief description of RRT will be provided. RRT explores the space by growing a tree *G*=(*V*, *Con*) from *x*_*start*_, where *V* is a set of vertices, and *Con* represents the connections between the vertices. As the tree expands towards the goal point *x*_*goal*_, a feasible path is generated. The tree’s growth occurs through multiple iterations. In each iteration, a random sample *x*_*rand*_ is selected from *X*_*free*_, and the closest vertex *x*_*nearest*_ in *V* is identified.

**Algorithm 2:**
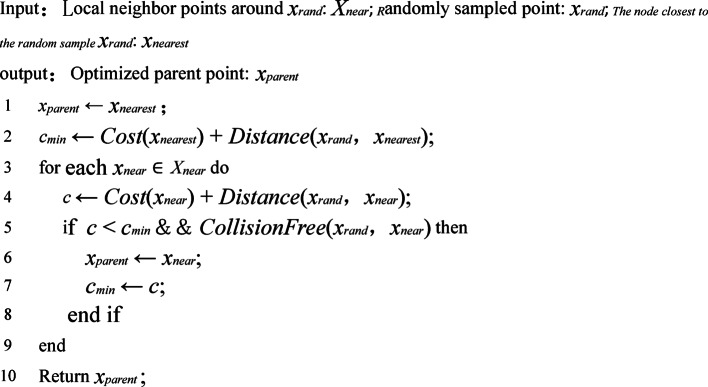
Choose parent.

**Algorithm 3:**
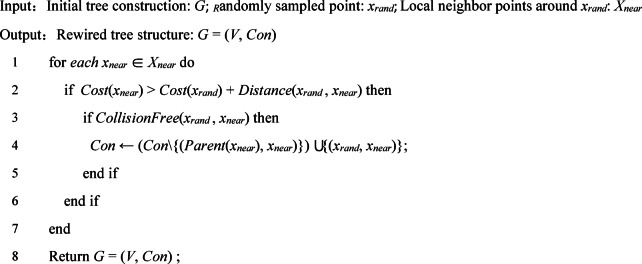
Rewire.

**Fig. 6:**
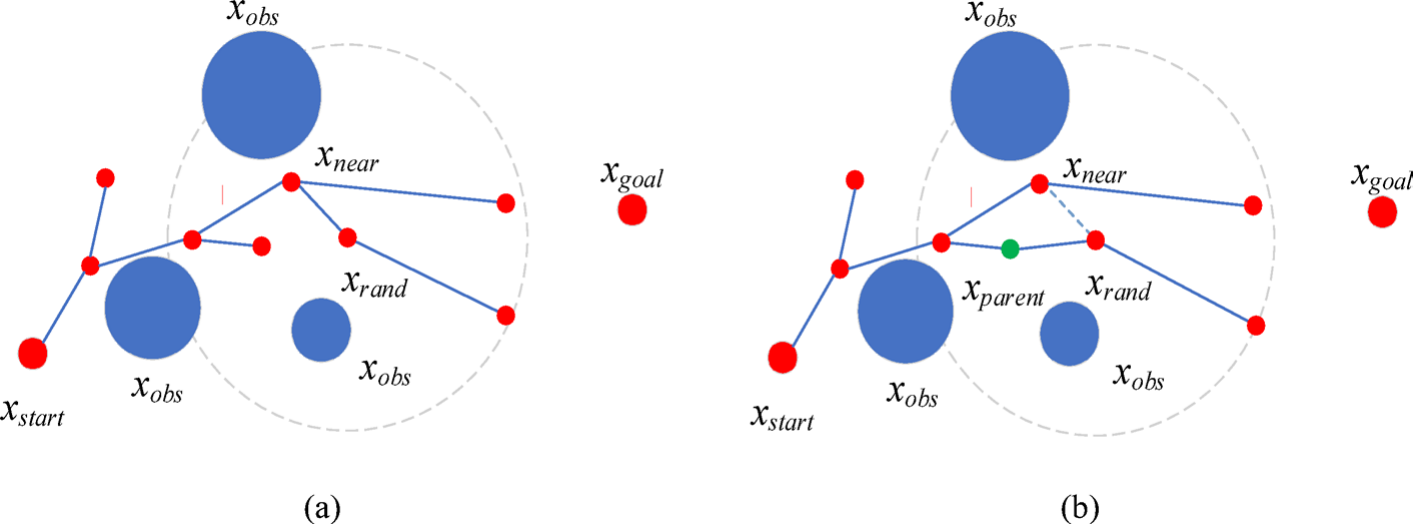
Schematic diagram of RRT* algorithm (**a**) Initial path generated by RRT (**b**) RRT*-Reselect parent node. *x*_*start*_ represents the initial node, *x*_*obs*_ represents the obstacle, *x*_*goal*_ represents the target node, *x*_*rand*_ represents the newly generated random node, *x*_*parent*_ represents the re-selected parent, and *x*_*near*_ represents the initial parent of *x*_*rand*_. The dashed circle represents the neighborhood range of *x*_*rand*_.

The PC-RRT* algorithm proposed is derived from the RRT* algorithm. The primary distinctions between RRT* and RRT are the optimization modules, ChooseParent, and Rewire. The purpose of ChooseParent, as described in Algorithm 2 and Fig. 6a, is to reduce the cost of the path from *x*_*start*_ to *x*_*rand*_ by searching for *X*_*near*_ (a set of vertices within a hypersphere of radius *R*_*near*_ centered at *x*_*rand*_). During this search, the node *x*_*parent*_ is selected from *X*_*near*_ to replace the candidate parent node *x*_*nearest*_ of *x*_*rand*_, thus lowering the cost of the path from *x*_*start*_ to *x*_*ran*d_.

**Fig. 7:**
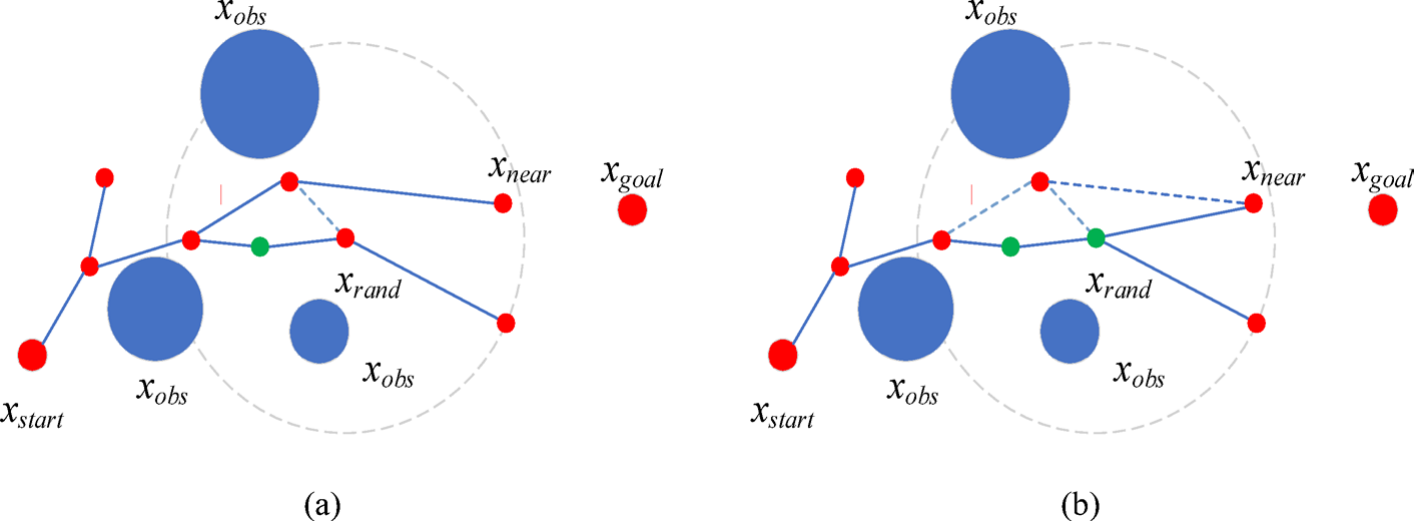
Schematic diagram of RRT* algorithm (**a**) RRT*-Reselect parent node (**b**) RRT*- Reconnect. *x*_*start*_ represents the initial node, *x*_*obs*_ represents the obstacle, *x*_*goal*_ represents the target node, *x*_*rand*_ represents the newly generated random node, *x*_*parent*_ represents the re-selected parent, and *x*_*near*_ represents one of the nodes of the initial path. The dashed circle represents the neighborhood range of *x*_*rand*_.

Parent node reselection is subsequently followed by connection re-establishment, as depicted in Fig. 7. In the rewire procedure, the paths from *x*_*start*_ to the vertices in *X*_*near*_ are optimized by *x*_*rand*_. For any point *x*_*near*_ in *X*_*near*_, if replacing its parent node with *x*_*rand*_ reduces the path cost from *x*_*start*_ to *x*_*near*_, the replacement will occur. As described in Algorithm 3, the Rewire procedure ensures asymptotic optimality by modifying the tree’s connection relationships, as shown in Fig. 7(b).

### Path pruning

**Fig. 8:**
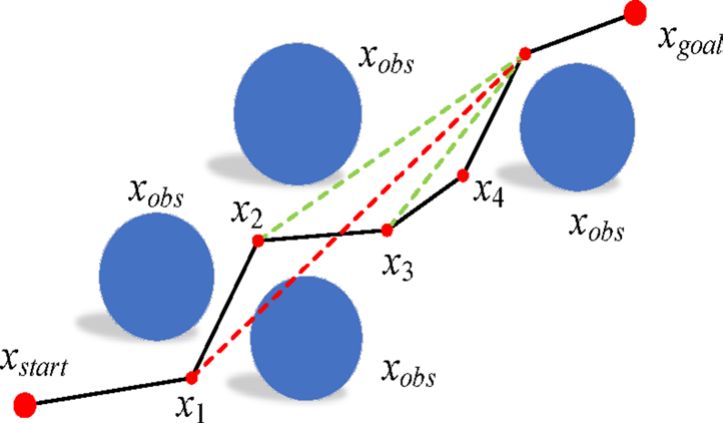
Geometric diagram of removing redundant nodes in the initial path. *x*_*start*_ represents the initial node, *x*_*obs*_ represents the obstacle, and *x*_*goal*_ represents the target node. The green dashed line represents that the path of the re-selected parent node is feasible, while the red dashed line represents that the path of the re-selected parent node is not feasible.

In the PC-RRT* algorithm, path pruning is crucial for optimizing the tree’s paths. It evaluates and removes redundant paths to bring the generated paths closer to the optimal solution, as described in Fig. 8. The main steps are as follows: First, start from the target node and backtrack to the starting node, calculating each node’s actual cost (typically the path length) from the start node and the straight-line distance from the node to the target node to evaluate path quality. Examine each node’s neighbors. Second, if a lower path cost is found via a neighbor, update the neighbor as the current node’s parent and adjust the path cost. Finally, continue updating in reverse until reaching the start node. After completing the backtracking, update the tree structure and path costs, which typically results in a shorter and more optimal path^39^.

### Path smoothing

A non-smooth path can cause the robotic arm to frequently adjust its motion state, leading to increased wear on joints and drive systems, reduced motion speed and efficiency, and higher energy consumption. To address this, the cubic, quartic, and quintic interpolation polynomials were tested, and it was found that the path generated by the quartic interpolation polynomial provided the best balance of shortness and smoothness. As a result, PC-RRT* employs a quartic interpolation polynomial for node interpolation, which helps create smoother paths and mitigate these issues.

To ensure the robot’s obstacle avoidance performance, the given nodes must be processed. If the distance between a node and an obstacle is below the threshold, the node’s position is adjusted to increase the distance from the obstacle, followed by quartic interpolation polynomial planning. Additionally, when fitting three-dimensional nodes, extra interpolation points are added to ensure that they lie within the specified constraint plane.

The detailed steps are as follows: First, by using the node coordinates from the unfitted PC-RRT* algorithm, *y* is regarded as the dependent variable and *x* as the independent variable. Assuming that the equation is mathematically satisfied *f*_*i*_(*x*)=*a*_*i*_*x*^4^+*b*_*i*_*x*^3^+*c*_*i*_*x*^2^+*d*_*i*_*x*+*e*_*i*_. To determine the coefficients *a*_*i*_, *b*_*i*_, *c*_*i*_, *d*_*i*_, and *e*_*i*_ of the fitting path, assuming having *n* data points, the coefficients of the quartic polynomial can be solved by using the end function values, the continuity of the first derivative, the continuity and boundary conditions of the second derivative, and the continuity and boundary conditions of the additional third derivative. Secondly, given that the node in question is three-dimensional, the plane equation *Ax* + *By*+*Cz* + *D* = 0, derived from Rodrigues’ rotation formula, can be utilized to substitute the fitted *x* and *y* values to determine the corresponding *z* value. Applying this interpolation method yields a smooth path, ensuring that the manipulator operates smoothly within the specified range.

### Predictive assessment method of planar solution feasibility

PC-RRT* is suitable for most scenarios. However, it has limitations when handling complex situations, such as those involving pipelines or dense obstacles. In three-dimensional path planning, accurately determining the existence of feasible planar solutions in advance is a challenge. A predictive method called local bounding detection is presented. This method preliminarily assesses the likelihood of the existence of planar solutions. The judgment results provide a probabilistic reference value.

A rapid determination rule based on the analysis of local spatial obstacle distribution is proposed. The core idea is that if local obstacles closely surround both the starting point and the goal point, the probability of finding a feasible planar solution at the global scale will significantly decrease.

**Fig. 9:**
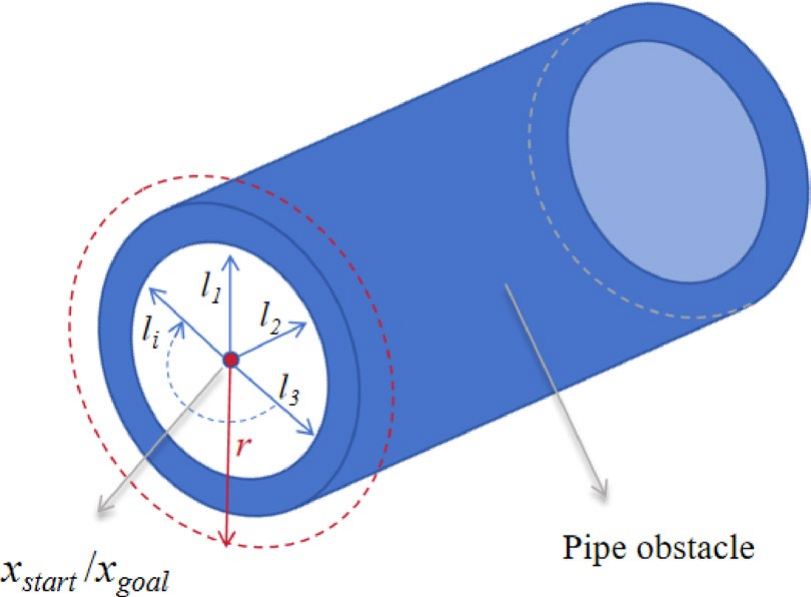
Pipe schematic diagram.

Specifically, a predefined detection radius, *r* (for example, set to *r* = 0.5 m), is defined with the starting point (*x*_*start*_) and the goal point (*x*_*goal*_) as its centers. For the local circular area **ℬ** (*x*_*c*_, *r*) centered at point *x*_*c*_∈{*x*_*start*_, *x*_*goal*_}, the shortest distance from all obstacle surfaces within that area to *x*_*c*_ is calculated, denoted as the set {*l*_*i*_^(c)^}. Two types of spatial constraint features and their determination conditions are defined as follows:

1. Pipe/cavity structure features: All distances *l*_*i*_^(c)^ from points *x*_*c*_ within the region **ℬ** (*x*_*c*_, *r*) to the obstacles are less than *r*, and the schematic is shown in Fig. 9. The specific expression is given by Eq. (14).

14 $$\forall i,{\text{ }}l_{i}^{{(c)}}<r$$

where the point *x*_*c*_ is classified as being within a structure (such as a pipe or cavity) that is surrounded by obstacles. The schematic is shown in Fig. 9.

**Fig. 10:**
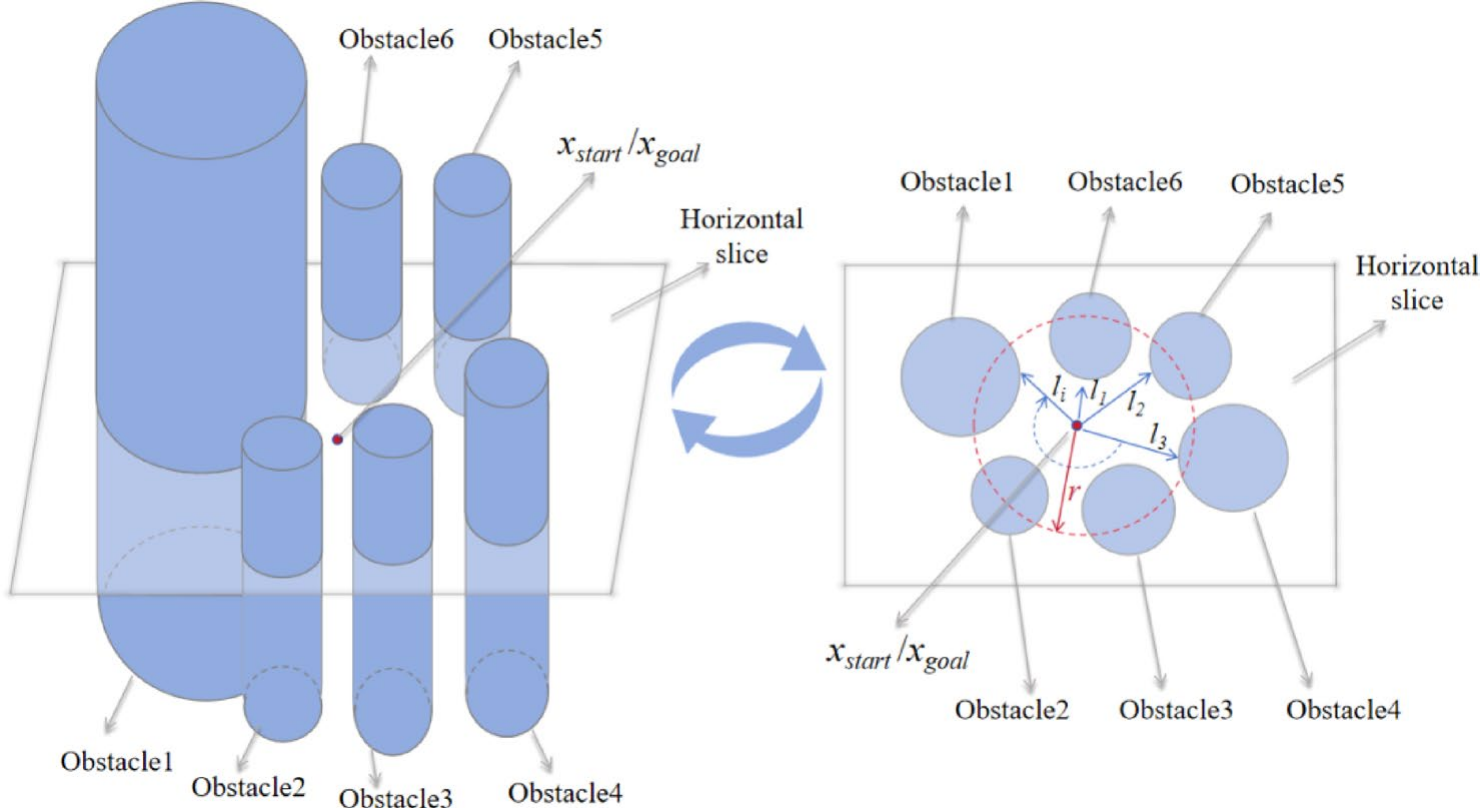
Dense obstacles schematic diagram.

2. Dense obstacle environment features: If the proportion of distances *l*_*i*_^(c)^ that are less than *r* (for points *x*_*c*_ within **ℬ**) exceeds a specific threshold (e.g., 80%), the region is characterized as having a dense obstacle concentration. This criterion is quantified by Eq. (15).

15 $$\frac{{\left| {\left\{ {l_{i}^{{(c)}}:l_{i}^{{(c)}}<r} \right\}} \right|}}{{\left| {\left\{ {l_{i}^{{(c)}}} \right\}} \right|}} \geqslant 0.8$$

where the numerator represents the number of elements in the set {*l*_*i*_^(c)^} with distances less than *r*, while the denominator indicates the total number of elements in the set {*l*_*i*_^(c)^} within the detection range. When the condition in Eq. (15) is satisfied, it can be concluded that the point *x*_*c*_ is located in a dense space of obstacles, with a high likelihood of restricting the accessibility of paths. A schematic is shown in Fig. 10.

The criterion is specifically defined as follows: the rule is triggered if either *x*_*start*_ or *x*_*goal*_ meets any of the restricted conditions described above, namely, possessing pipeline/cavity characteristics or being located in a dense obstacle environment. The system can determine that there is a high probability of no feasible planar solution in the current environment.

Local bounding detection effectively addresses the majority of situations, but judgment deviations may still occur in extremely complex scenarios. By distinguishing between the two types of spatial constraint patterns, this approach maintains sensitivity to enclosed structures while enhancing adaptability to dense environments. It provides an effective basis for predicting whether to initiate the PC-RRT* algorithm.

### Numerical simulations

#### Comprehensive simulation and analysis of the PC-RRT* algorithm

When the manipulators need to perform repetitive work to meet certain industrial requirements, to save energy, they can find the optimal plane by traversing different planes and then perform repetitive work. Therefore, the influence of the angle interval on high-quality path finding is proposed. The specific procedure is as follows: a reference plane is established based on the straight-line distance between the starting point and the target point. The reference plane is then rotated at specified angular intervals to identify the optimal plane. The selection of the optimal plane prioritizes obstacle avoidance and minimizes path length.

**Fig. 11:**
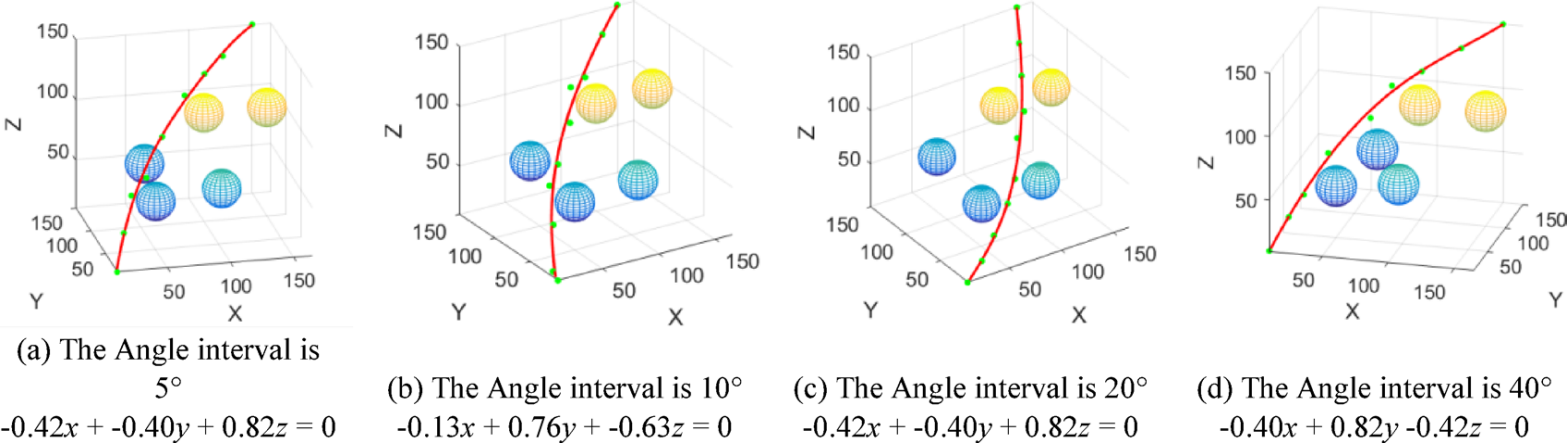
High-quality path planning diagram obtained at different angle intervals.

Since each different angular interval results in a corresponding optimal plane, the subsequent simulation aims to investigate the impact of the angular interval size on the path quality generated on the optimal plane. Under the condition that the starting point, target point, and obstacle environment remain unchanged, this experiment is designed with angular intervals of 5°, 10°, 20°, and 40° to cover the 360-degree range. For each angular interval, the corresponding optimal plane is identified, and the path planning on the optimal plane is illustrated in Fig. 11. The analysis in Fig. 11 indicates that the minimum search path length obtained is shorter when the Angle interval of the search plane is smaller.

**Fig. 12:**
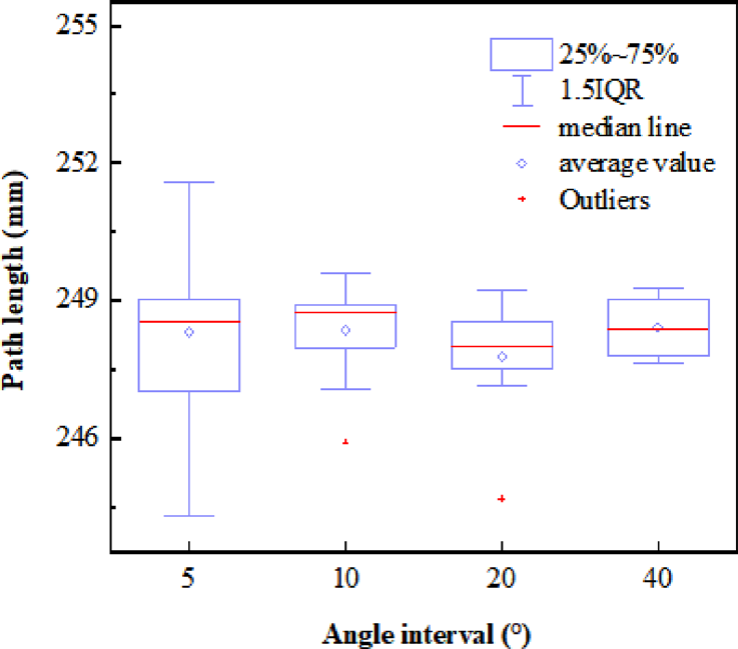
Statistical results of path length at different angle intervals.

To mitigate the impact of randomness on the experimental results, multiple paths planning of obstacles was carried out with Angle intervals of 5°, 10°, 20°, and 40°, respectively, with the results shown in Fig. 12. The mean and median values for each angular interval range from 248 to 249. To identify the optimal plane for search, the plane equation corresponding to the last 25% quantile with the highest frequency of shortest path occurrences within each angular interval is computed, and the most frequent plane equation is determined, as shown in Table 1.

**Fig. 13:**
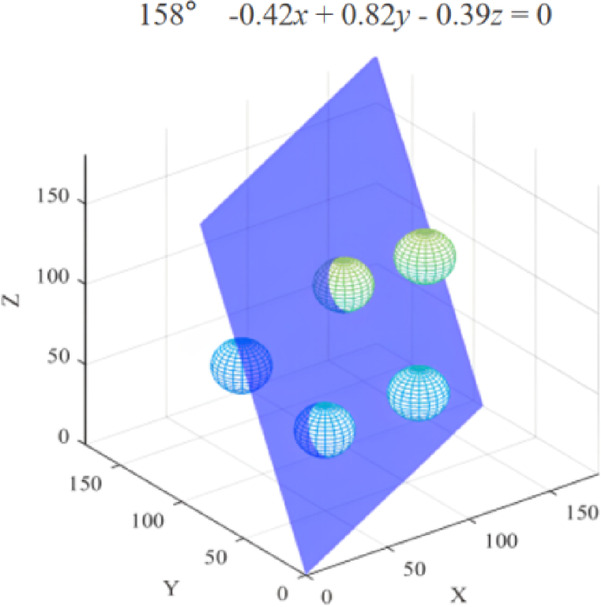
Optimal plane cutting obstacle display diagram.

Based on the figure above, the experiment will be repeated at 1° angular intervals within the ranges of 35° to 45° and 155° to 165°. This approach aims to identify the optimal plane by calculating the path lengths and to investigate the potential for discovering a shorter path. As illustrated in Fig. 13, starting from the reference plane described by the Eq. (0.13*x* − 0.76*y* + 0.63*z* = 0), a new plane equation (-0.42*x* + 0.82*y* − 0.39*z* = 0) is derived by rotating 158°. This optimal plane serves as a constraint for the path nodes generated by the RRT* algorithm, thereby mitigating path indeterminacy and randomness, to minimize both the path length and the number of nodes.

**Table 1:** Planes with the most occurrences of the last 25% quantile.

Angle of the plane equation	Plane equation (Ax + By+Cz + D = 0)	APL/mm	Number of occurrences in the last 25% quantile
160°	-0.40*x* + 0.82*y* + -0.42*z* = 0	246.007	3
40°	-0.42*x* + -0.40*y* + 0.82*z* = 0	246.828	3

### Validations in different test environments

#### Complex obstacle environment 1

To validate the universality of the PC-RRT* algorithm across various environments, path planning tests were conducted in different obstacle scenarios. In the subsequent experiments, it is assumed that the robotic arm does not need to identify the optimal plane, but instead focuses on finding a practical solution. The constrained plane used in this case is randomly selected. Furthermore, the PC-RRT* algorithm also demonstrates superior performance in terms of path length in this case.

**Fig. 14:**
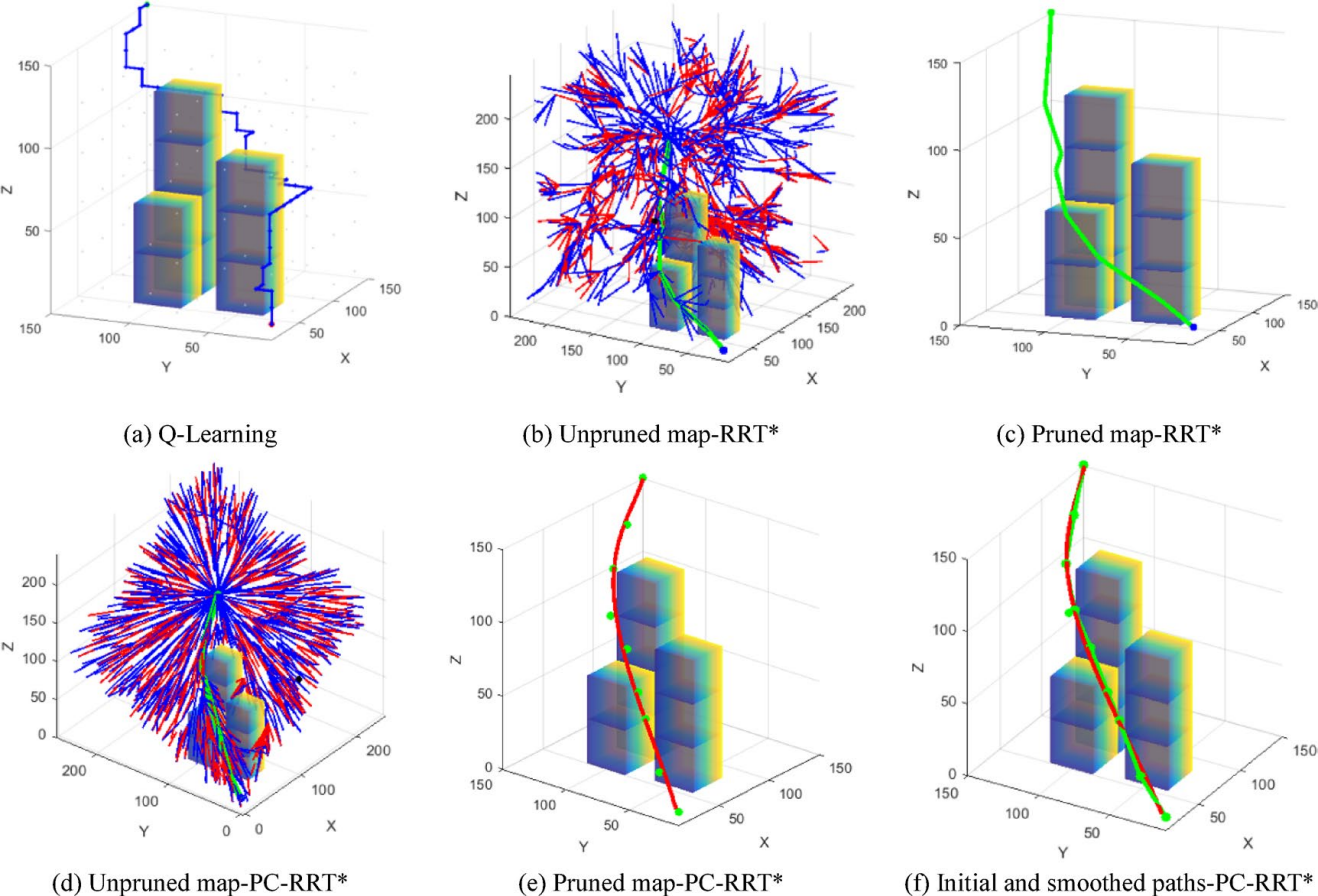
Path planning comparison of different algorithms. (**a**) Q-Learning, (**b**) Unpruned map-RRT*, (**c**) Pruned map-RRT*, (**d**) Unpruned map-PC-RRT*, (**e**) Pruned map-PC-RRT*, (**f**) Initial and smoothed paths-PC-RRT*.

The path planned by the Q-learning algorithm is shown in Fig. 14a. Q-learning is one of the most classic and important model-free algorithms in the field of reinforcement learning. Its core idea is to allow the agent to interact with the environment, learn autonomously, and find one of the optimal strategies to reach the goal state from any given state. As illustrated, Fig. 14b shows the unpruned graph of the RRT* algorithm, while Fig. 14c displays the pruned graph of the RRT* algorithm. Similarly, Fig. 14d represents the unpruned graph of the PC-RRT* algorithm, and Fig. 14e represents the pruned graph of the PC-RRT* algorithm. Furthermore, Fig. 14(f) compares the initially planned path (green line) and the fitted smooth path (red line) for PC-RRT*. Graphical results show that the PC-RRT* algorithm also significantly reduces path length and improves trajectory smoothness under varying obstacle shapes.

**Fig. 15:**
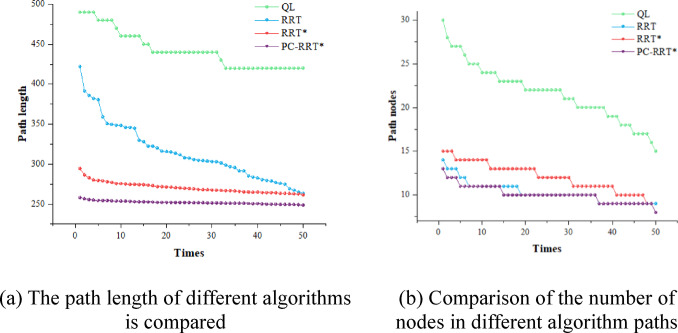
Comparison of different algorithms.

To assess the superiority of the proposed algorithm, 50 simulation experiments were conducted in the described obstacle environment using the RRT, RRT*, Q-learning, and PC-RRT* algorithms. The path metrics of these algorithms were analyzed (see Fig. 15). The results demonstrate that the PC-RRT* algorithm exhibits substantial improvements in path length optimization and node efficiency compared to the other algorithms. In the subsequent Table 5, the node data were recorded from the end-effector of the robotic arm using a laser tracker. Considering the inherent measurement errors in practical experiments, three measurements were taken for each data point, and the average value was subsequently computed.

For the path planning of the measuring manipulator, when the obstacle environment remains fixed, the system will carry out multiple repeated measurements on a specific object to be tested, necessitating only a single path planning. In this scenario, a shorter path length and a reduced number of nodes are prioritized over computational efficiency in planning. The specific evaluation procedure follows the description in the subsection “Spatial High-Quality Path Decision” within the “Overview of Planar Constraint PC-RRT Method” section. The corresponding evaluation metrics are Eqs. (1) and (2) as given in that subsection. A lower value of *E* indicates superior comprehensive performance of the algorithm. The metric *E* and respective rankings are provided in the penultimate and final columns of Table 2. PC-RRT* achieves high-quality performance with the smallest *E*-value, consequently attaining the highest ranking in the weighted evaluation.

As shown in Table 2, the PC-RRT* algorithm proposed in this paper demonstrates significant improvement in robot arm path planning compared to the traditional RRT* algorithm, reflecting its superior performance and efficiency in planning complex environments and optimizing trajectories. The APL of this algorithm is 250.29 mm, with a standard deviation of 1.69 mm, and an average of 10.6 path nodes. Compared to the traditional RRT* algorithm, the PC-RRT* algorithm reduces the APL by 10.99% and the number of nodes by 19.33%. When compared to the RRT* algorithm, the PC-RRT* algorithm reduces the APL and the number of nodes by 28.92% and 5.69%, respectively. Compared to the Q-learning algorithm, the PC-RRT* algorithm reduces the APL by 43.48% and the number of path nodes by 51.15%. The standard deviations of path length and path nodes for the PC-RRT* algorithm are significantly lower than those for both the RRT, RRT*, and Q-learning algorithms. The PC-RRT* algorithm not only reduces path length and node count but also exhibits high stability.

**Table 2:** Comparison of different algorithm metrics.

	APL/mm	Mean planning time/s	Path length standard deviation	Average number of nodes	Path node standard deviation	Weighted Normalized exponent	Weighted ranking
RRT	352.14	0.03	70.52	11.24	2.39	0.33	3
RRT*	281.19	31.36	14.92	13.14	2.28	0.22	2
QL	442.8	58.73	23.22	21.7	3.28	1	4
PC-RRT*	250.29	50.24	1.69	10.6	1.62	0.09	1

### Complex obstacle environment 2

**Fig. 16:**
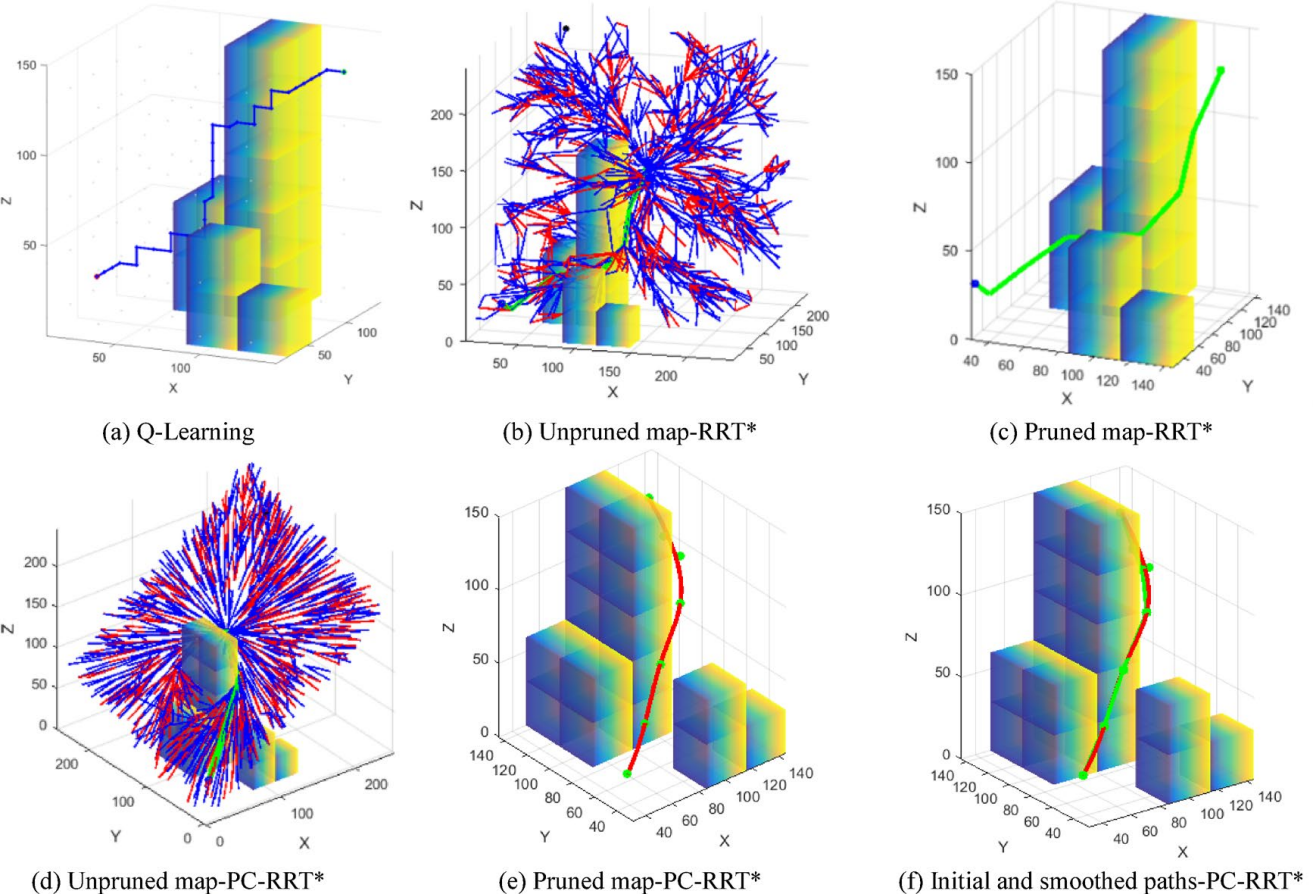
Path planning comparison of different algorithms. (**a**) Q-Learning, (**b**) Unpruned map-RRT*, (**c**) Pruned map-RRT*, (**d**) Unpruned map-PC-RRT*, (**e**) Pruned map-PC-RRT*, (**f**) Initial and smoothed paths-PC-RRT*.

Similarly, to verify the applicability of the PC-RRT* algorithm in diverse environments, additional tests were performed in various obstacle scenarios, as shown in Fig. 16.

The path planned by the Q-learning algorithm is shown in Fig. 16a. As illustrated, Fig. 16b shows the unpruned graph of the RRT* algorithm, while Fig. 16c shows the pruned graph of the RRT* algorithm. Similarly, Fig. 16d represents the unpruned graph of the PC-RRT* algorithm, and Fig. 16e represents the pruned graph of the PC-RRT* algorithm. Furthermore, Fig. 16f compares the initially planned path (green line) and the fitted smooth path (red line) for PC-RRT*. Graphical results show that the PC-RRT* algorithm also significantly reduces path length and improves trajectory smoothness under varying obstacle shapes.

**Fig. 17:**
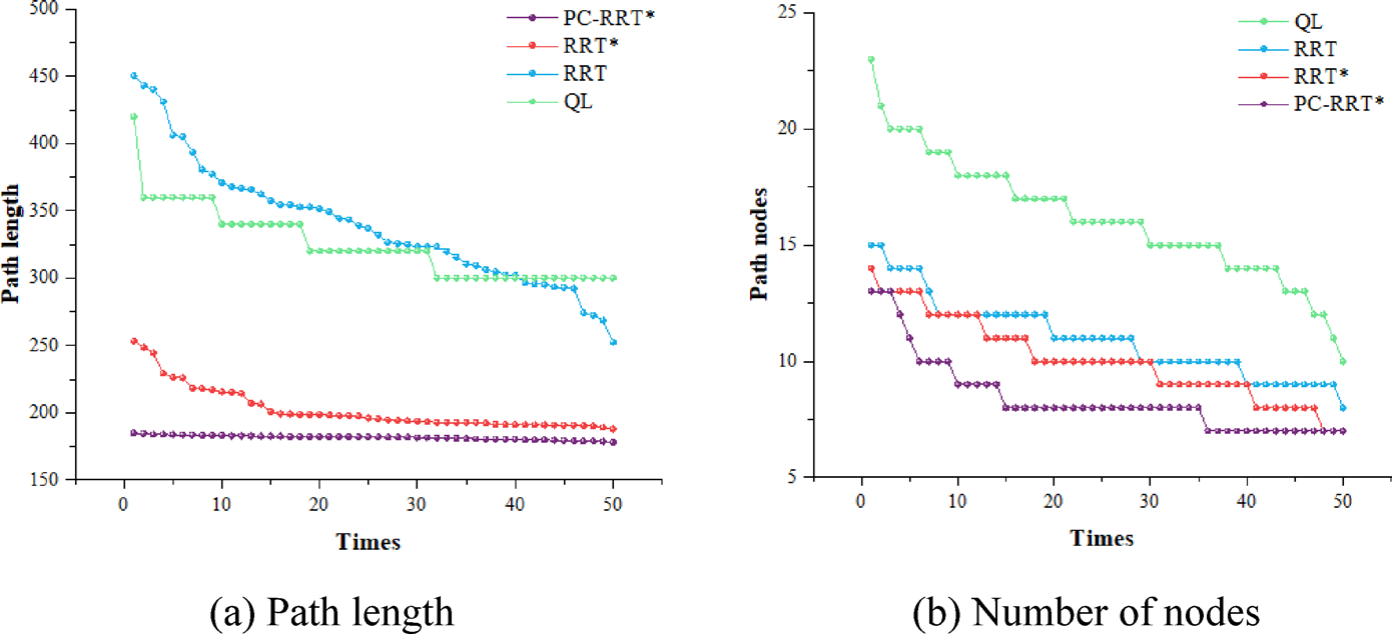
Comparison of different algorithms. (**a**) Path length, (**b**) Number of nodes.

Similarly, to evaluate the efficacy of the proposed algorithm, 50 simulation experiments were conducted using the RRT, RRT*, Q-learning, and PC-RRT* algorithms within the same obstacle environment, and the path metrics for each algorithm were analyzed (see Fig. 17). The results demonstrate that the PC-RRT* algorithm exhibits substantial improvements in path length optimization and node efficiency compared to the other algorithms. For the measuring manipulator, when the measurement scenario is fixed, a shorter measurement path with fewer nodes is more advantageous than reducing the algorithm planning time. Weight normalization evaluation for each algorithm is performed using Eqs. (1) and (2). Table 3 provides a comprehensive comparative analysis.

Table 3 indicates that the PC-RRT* algorithm outperforms the traditional RRT* algorithm in robot arm path planning. Specifically, the APL achieved by the PC-RRT* algorithm is 181.76 mm with a standard deviation of 1.58, and the average number of nodes is 8.4 with a standard deviation of 1.62. Compared to the traditional RRT* algorithm, the PC-RRT* algorithm reduces the APL by 10.31% and the number of nodes by 16.5%. When compared to the RRT* algorithm, the PC-RRT* algorithm reduces the APL and the number of nodes by 46.78% and 23.64%, respectively. Compared to the Q-learning algorithm, the PC-RRT* algorithm reduces the APL by 43.97% and the number of path nodes by 47.96%. The metric *E* and respective rankings are provided in the penultimate and final columns of Table 3. PC-RRT* achieves high-quality performance with the smallest *E*-value, consequently attaining the highest ranking in the weighted evaluation. Furthermore, the PC-RRT* algorithm demonstrates significantly lower standard deviations in both path length and node count, highlighting its enhanced stability in path planning.

**Table 3:** Comparison of different algorithm metrics.

	APL/mm	Mean planning time/s	Path length standard deviation	Average number of nodes	Path node standard deviation	Weighted Normalized exponent	Weighted ranking
RRT	341.51	0.045	46.68	11	1.71	0.7	3
RRT*	202.66	36.78	15.99	10.06	1.78	0.22	2
QL	324.4	43.92	25.96	16.14	2.66	0.93	4
PC-RRT*	181.76	48.14	1.58	8.4	1.62	0.1	1

**Fig. 18:**
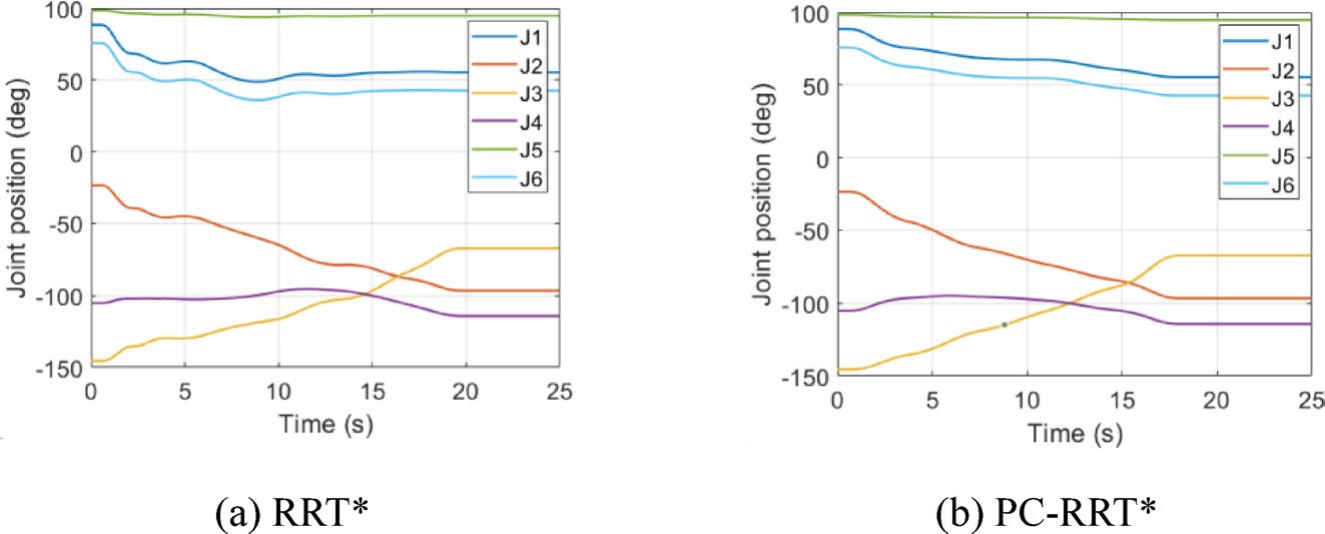
Joint Angle variation in obstacle environment 1. (**a**) RRT*, (**b**) PC-RRT*.

**Fig. 19:**
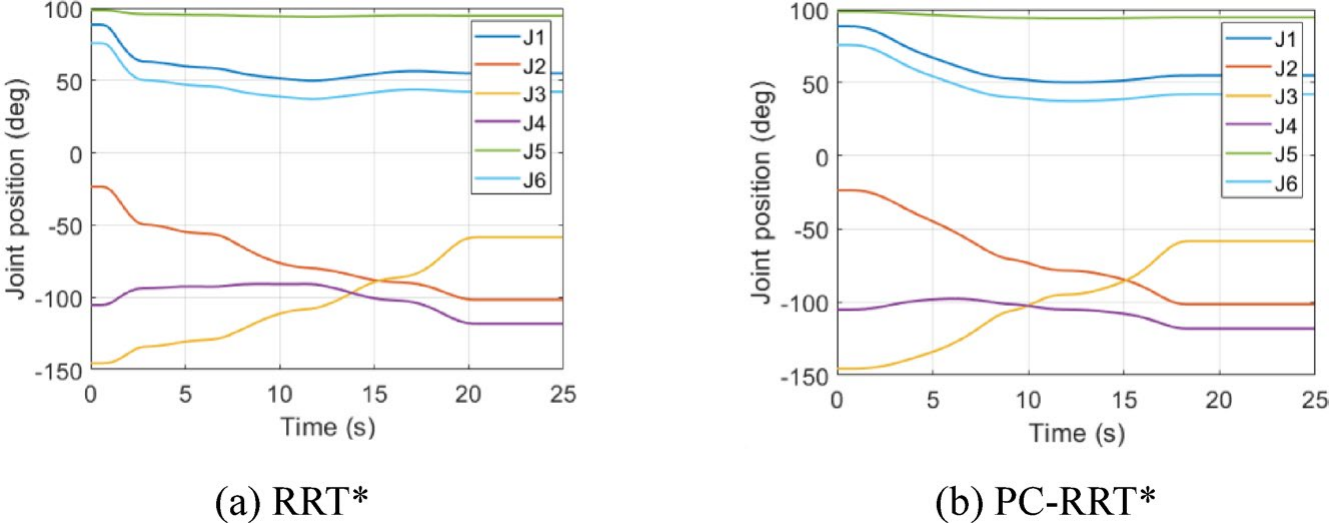
Joint angle variation in obstacle environment 2. (**a**) RRT*, (**b**) PC-RRT*.

Both the RRT* and PC-RRT* algorithms complete the trajectory planning. As shown in Figs. 18 and 19, in terms of detail, the joint angle variation curve during the robotic arm’s path planning with the RRT* algorithm is less smooth compared to that of the PC-RRT* algorithm. In summary, the proposed algorithm demonstrates clear advantages in robot arm path planning, including shorter path lengths and smoother trajectories. This approach effectively reduces the path length and the associated standard deviation, enhances efficiency, and conserves energy. This approach effectively enhances path smoothness, reducing joint wear, and extending the robot arm’s service life. These characteristics endow the algorithm with high practical value and offer a valuable reference for research in robot arm path planning. This method is also applicable to other 3D algorithms, providing an effective approach to improving planning efficiency. Experimental results validate the effectiveness and performance advantages of the proposed algorithm.

### Experiments and analysis

#### Experimental setup

**Fig. 20:**
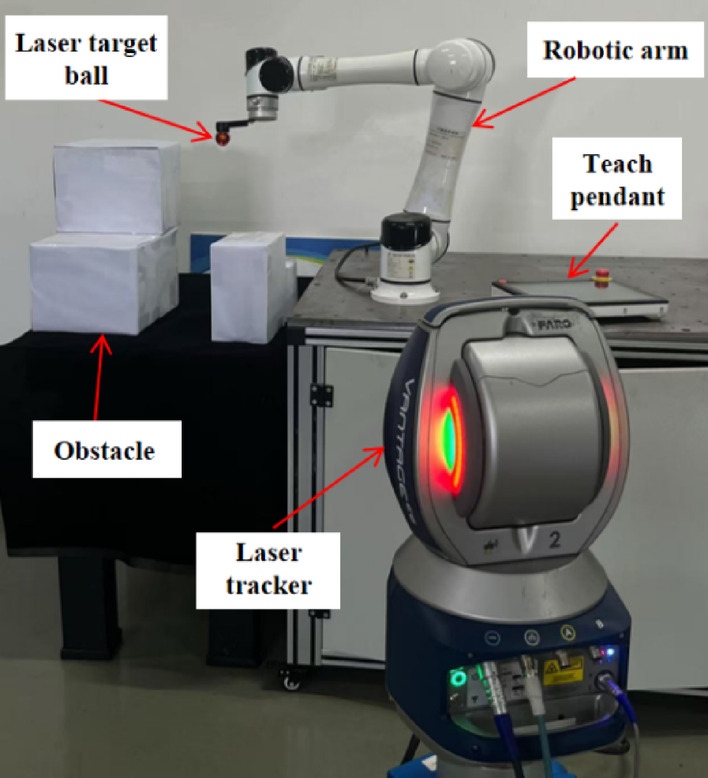
Experimental system test chart.

To demonstrate the application advantages of the improved PC-RRT* algorithm in the measurement process, a path planning experimental system has been designed. This system employs a laser tracker to measure nodes during the manipulator’s operation and to record experimental data. It primarily consists of a six-degree-of-freedom robotic arm, a teaching device, a laser tracker, a laser target ball, and an obstacle environment, as depicted in Fig. 20. During the experimental trials, the end-effector position of the robotic manipulator was measured utilizing the Faro laser tracker.

The selected measuring robot has an effective working radius of 815 mm and a joint range of ± 360°, with the DH parameters shown in Table 4. The DH parameters are defined as follows: ***α***_*i*−1_ is the twist angle about *X*_*i*−1_ from *Z*_*i*−1_ to *Z*_*i*_, ***a***_*i*−1_ is the link length along *X*_*i−1*_ from *Z*_*i*−1_ to *Z*_*i*_, *d*_*i*_ is the link offset along *Z*_*i*_, and *θ*_*i*_ is the joint angle about *Z*_*i*_ (the actuated configuration variable). The probe is attached to the end of the arm using a connector. When the probe is added, it alters the arm’s end position, necessitating calibration to determine the new endpoint. The experimental procedure involves setting up the simulation environment in MATLAB, where the simulation path is sent to the controller as joint angles through Socket and WebSocket protocols. This enables the robot to follow the planned path and reach the target position.

**Table 4:** DH parameter of the AR5 measuring robot.

Joint	α_i−1_(rad)	a_i−1_(mm)	θ_i_(rad)	d_i_(mm)
1	0	0.00	*θ*_1_	100.15
2	$\pi\mathrm{/2}$	0.00	*θ*_2_	108.27
3	0	426.76	*θ*_3_	0.00
4	0	395.09	*θ*_4_	0.00
5	-$\pi\mathrm{/2}$	0.00	*θ*_5_	97.10
6	-$\pi\mathrm{/2}$	0.00	*θ*_6_	82.57

### Experimental data analysis

**Fig. 21:**
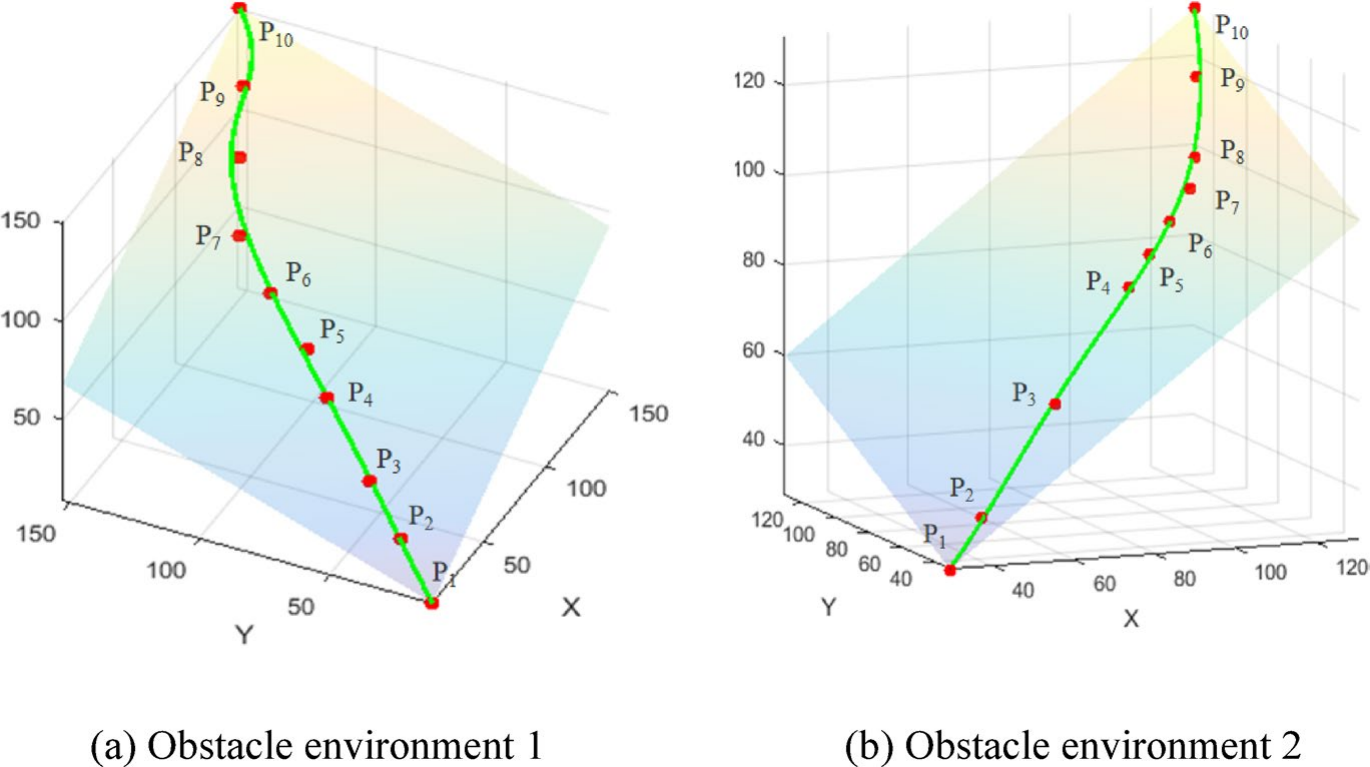
Fitting curve graph of measurement points. (**a**) Obstacle environment 1, (**b**) Obstacle environment 2.

To enhance the accuracy of the experimental data, three experiments were performed in each of the complex obstacle environments 1 and 2. Tables 5 and 6 present the ten sets of measured point data collected from experiments. The distribution of these data points demonstrated that the robotic arm effectively avoided obstacles. To determine if these measured points lie on the same plane, Line fitting and plane fitting were conducted using the calculated average points *P*_*i*_ derived from the data in Tables 5 and 6. This is illustrated in Fig. 21.

As illustrated in Fig. 21, line fitting and plane fitting of the measured data points reveal that they are clearly situated on the same plane. This finding demonstrates that the PC-RRT* algorithm enables the robot arm to avoid obstacles during operation, maintaining the path approximately in the same plane to optimize the path and smooth the trajectory, thereby enhancing the robot arm’s efficiency in obstacle avoidance. In summary, the experimental results validate the effectiveness and performance advantages of the proposed algorithm, which demonstrates significant benefits in robot arm path planning.

**Table 5:** Complex obstacle environment 1.

	P1	P2	P3	P4	P5
Measured data 1	(8.909,10.112,8.861)	(22.184,28.755,24.895)	(33.405,46.365,39.078)	(50.785,71.087,59.550)	(61.747,83.695,71.120)
Measured data 2	(8.910,10.114,8.862)	(22.183,28.755,24.893)	(33.405,46.358,39.077)	(50.788,71.086,59.553)	(61.749,83.696,71.120)
Measured data 3	(8.911,10.117,8.862)	(22.183,28.751,24.893)	(33.400,46.364,39.075)	(50.787,71.087,59.552)	(61.750,83.701,71.118)
Mean value	(8.910,10.114,8.862)	(22.183,28.753,24.894)	(33.403,46.362,39.077)	(50.785,71.086,59.552)	(61.749,83.697,71.119)
	P6	P7	P8	P9	P10
Measured data 1	(72.171,102.646,84.788)	(83.375,120.477,98.354)	(106.729,131.643,116.652)	(127.892,140.531,132.920)	(150.544,152.719,151.601)
Measured data 2	(72.170,102.652,84.788)	(83.377,120.478,98.352)	(106.729,131.639,116.652)	(127.892,140.529,132.919)	(150.540,152.721,151.599)
Measured data 3	(72.167,102.653,84.785)	(83.380,120.477,98.354)	(106.727,131.642,116.652)	(127.890,140.532,132.919)	(150.541,152.719,151.598)
Mean value	(72.169,102.651,84.787)	(83.377,120.478,98.353)	(106.728,131.641,116.652)	(127.891,140.531,132.919)	(150.542,152.720,151.599)

**Table 6:** Complex obstacle environment 2.

	P1	P2	P3	P4	P5
Measured data 1	(28.950,29.663,29.031)	(39.752,36.676,38.834)	(63.637,51.150,59.953)	(88.350,67.100,81.885)	(95.193,71.583,87.970)
Measured data 2	(28.953,29.667,29.029)	(39.753,36.672,38.837)	(63.638,51.144,59.955)	(88.350,67.098,81.882)	(95.197,71.579,87.969)
Measured data 3	(28.951,29.667,29.031)	(39.752,36.670,38.835)	(63.638,51.144,59.954)	(88.350,67.093,81.887)	(95.196,71.580,87.971)
Mean value	(28.951,29.666,29.031)	(39.752,36.673,38.835)	(63.638,51.146,59.954)	(88.353,67.097,81.885)	(95.195,71.581,87.970)
	P6	P7	P8	P9	P10
Measured data 1	(101.998,76.091,94.105)	(108.818,80.634,100.241)	(113.108,88.211,105.522)	(122.569, 111.039, 119.049)	(130.593,131.292,130.795)
Measured data 2	(102.001,76.093,94.106)	(108.822,80.634,100.243)	(113.109,88.213,105.522)	(122.566, 111.043, 119.049)	(130.593,131.298,130.795)
Measured data 3	(102.000,76.092,94.108)	(108.819,80.633,100.244)	(113.107,88.215,105.523)	(122.569, 111.039, 119.051)	(130.594,131.295,130.797)
Mean value	(102.000,76.092,94.106)	(108.819,80.634,100.243)	(113.108,88.213,105.522)	(122.568, 111.040, 119.050)	(130.593,131.295,130.795)

## Conclusion

To address the issues of sub-optimal path quality and high randomness in the RRT* algorithm’s search process, the PC-RRT* algorithm is proposed—an enhanced RRT* variant that incorporates equidistant plane constraints. By constraining randomly generated nodes to a single plane and establishing consistent angular intervals to explore the 360-degree range, this approach helps identify the optimal plane in three-dimensional space, resulting in a shorter and smoother path. Numerical simulations and experimental analyses are conducted on three different maps to verify the effectiveness of the PC-RRT* method. The experimental results show that the APL of PC-RRT* is the highest quality among the three algorithms, with improvements of 37.71%, 10.71%, and 43.68% compared to the RRT, RRT*, and QL algorithms, respectively. Additionally, the PC-RRT* algorithm has the fewest nodes of the three algorithms, with reductions of 14.57%, 18.1%, and 49.79% compared to the RRT, RRT*, and QL algorithms. PC-RRT* applies to most scenarios, but exhibits limitations in complex situations, such as those involving pipelines and crowded obstacles. A more accurate method for assessing the existence of a planar solution will be developed in future work and integrated into PC-RRT*.

## Supplementary Information

Below is the link to the electronic supplementary material.

Supplementary Material 1

Supplementary Material 2

## Data Availability

The datasets used and analyzed during the current study available from the corresponding author on reasonable request.

## Notes

The code generated to visualize the results of this study is publicly available at 10.6084/m9.figshare.31400730 under a MIT license.
